# Supplementary material for: Single-cell RNA-sequencing data analysis reveals a highly correlated triphasic transcriptional response to SARS-CoV-2 infection
Source: Commun Biol. 2022 Nov 27;5:1302. doi: 10.1038/s42003-022-04253-4 (PMC9701238; doi:10.1038/s42003-022-04253-4)
Supplement: Supplementary file 2 — Supplementary Information [file 42003_2022_4253_MOESM2_ESM.pdf]

Supplementary information

Supplementary Table 1. Global statistics of the dataset used in the analysis.

Dataset	TPI	SRA accession	Total reads	Selected transcripts[1]	Transcripts per GEM	Selected GEMs[3]	Selected genes[*]
**Bronchial epithelial cells**	*Mock*	SRR13711613	604,127,582	92,488,540	1,878-16,810	16,531	
	1 dpi	SRR13711614	589,830,183	75,836,207	1,232-30,555	9,345	
	2 dpi	SRR13711615	653,390,536	90,806,792	1,752-20,120	12,472	
	3 dpi	SRR13711616	573,563,158	91,799,408	1,781-13,181	17,820	
			**2,420,911,459**	**350,930,947**		**56,168**	**9,913**
**Colon* organoids**	*Mock*	SRR12508049	294,735,427	71,838,718	1,030-47,489	4,024	
		SRR12508050	107,844,234	30,338,763			
	12 hpi	SRR12508052	304,892,652	79,187,463	1,149-46,952	3,992	
		SRR12508053	123,149,496	37,510,656			
	24 hpi	SRR12508055	307,339,187	79,260,961	1,232-31,513	4,407	
		SRR12508056	121,191,449	35,611,648			
			**1,259,152,445**	**333,748,209**		**12,423**	**7,582**
**Ileum* organoids**	*Mock*	SRR12508058	310,516,022	77,784,527	968-40,234	3,457	
		SRR12508059	115,263,635	35,651,119			
	12 hpi	SRR12508061	308,565,909	73,011,266	1,100-40,163	4,115	
		SRR12508062	116,802,746	33,699,484			
	24 hpi	SRR12508064	304,036,624	69,478,009	908-42,604	3,629	
		SRR12508065	115,167,592	33,137,814			
			**1,270,352,528**	**322,762,219**		**12,201**	**8,334**

[1]Only transcripts unambiguously attributed to a single gene and a single unique molecular identifier (UMI) were included in the analysis.
[2]Specifies the minimum and maximum number of transcripts required for a cell in the corresponding dataset to be included in the analysis.
[3]Total number of cells selected based on the number of transcripts.
[4]Total number of genes included in the analysis for each cell type. Selected genes required to expressed in a minimum of one thousand cells.
*Datasets from colon and ileum organoids comprised two separate files per experiment that were merged for subsequent analyses.
Abbreviations: GEM: Gel Bead-in Emulsion, SRA: Sequence read archive, TPI: time post-inoculation, dpi: days post-infection, hpi: hours post-infection.
Aggregated values for selected columns are shown in bold.

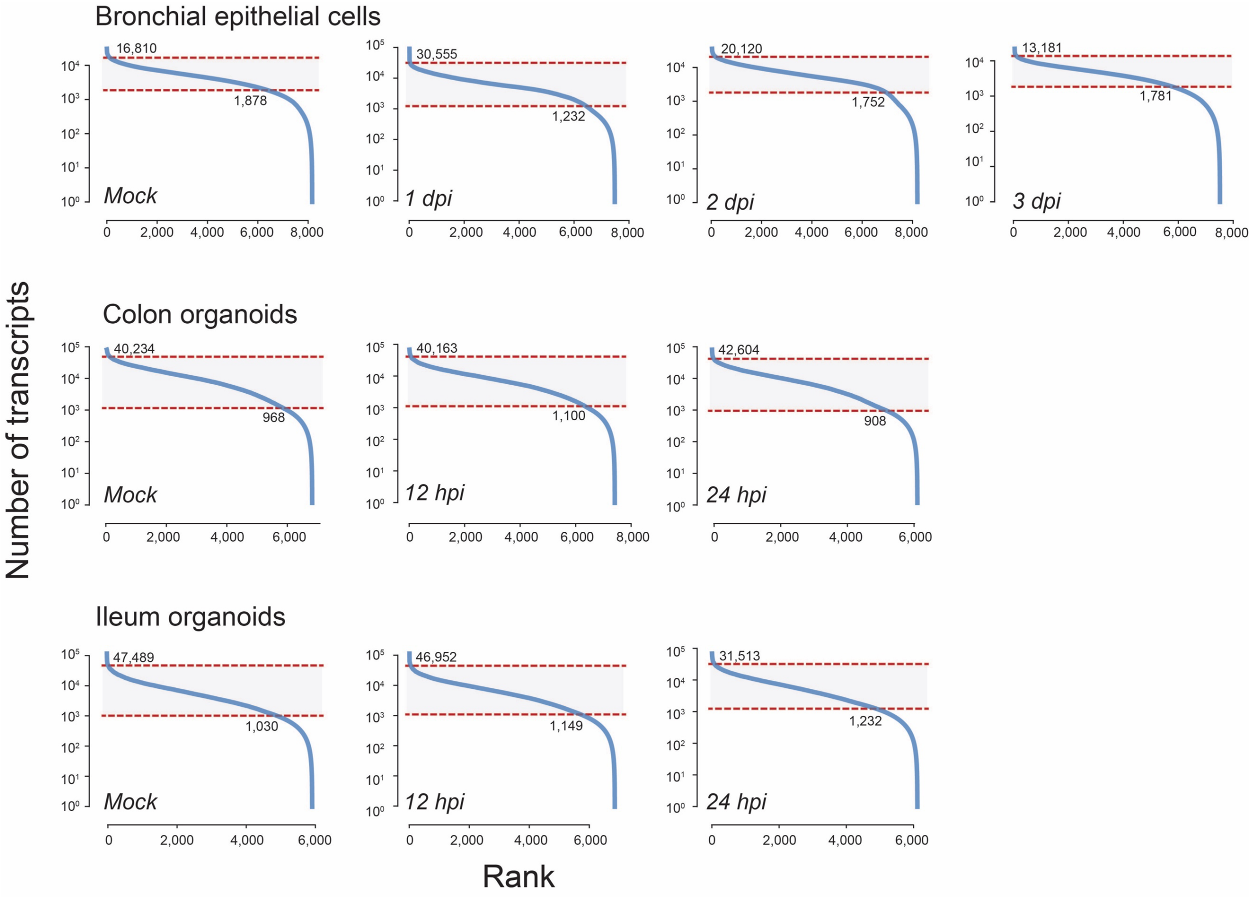

Supplementary Figure 1. Filtering of void GEMs and multiplets. GEMs were ordered with respect to the number of transcripts the local standard deviation of the $\log_{10}$ using window size of five datapoints. Upper and lower thresholds delimiting multiples and voids GEMS were determined using 1.5×IQR rule. The cells used in this study correspond to the grey area.

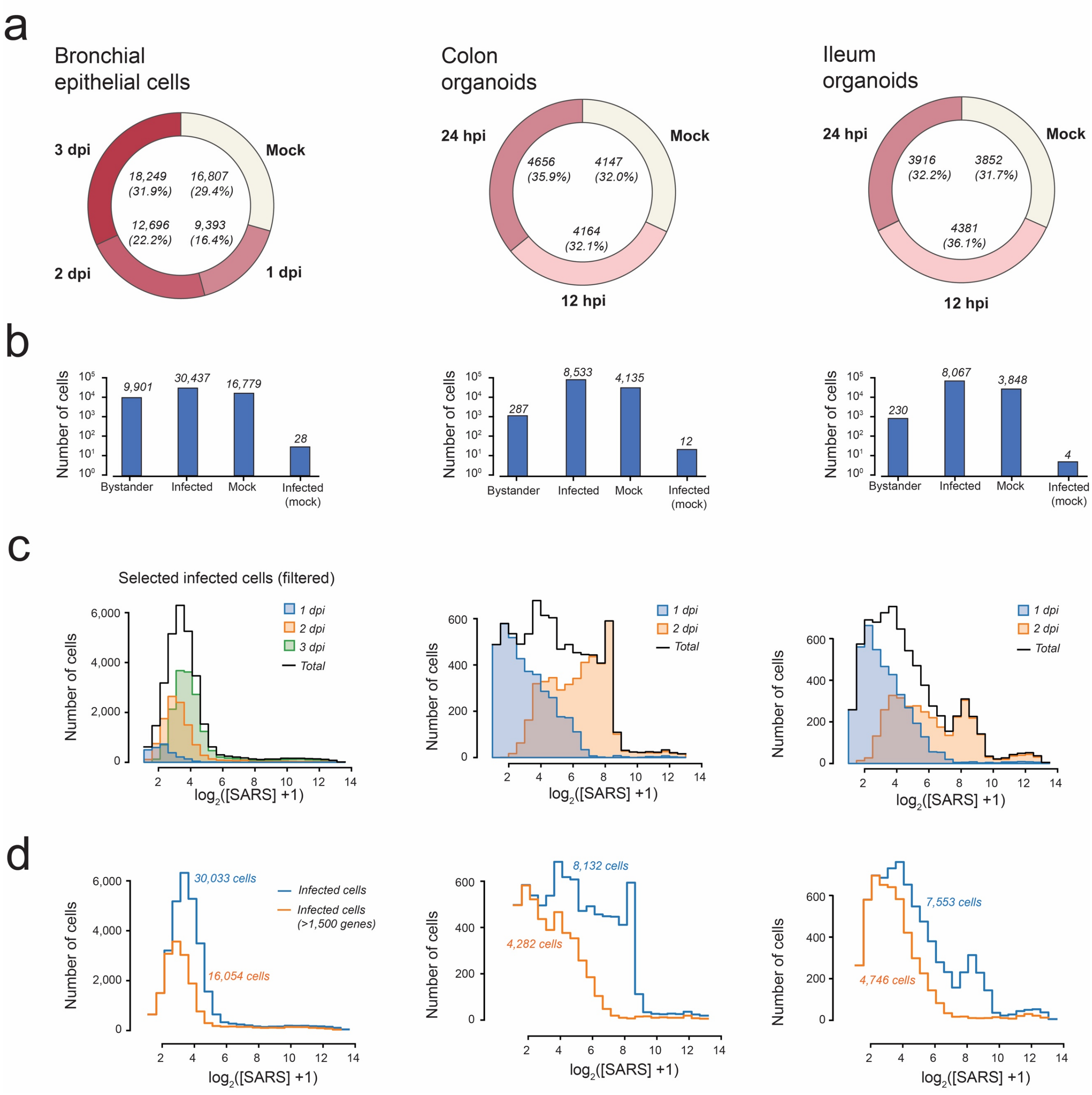

Supplementary Figure 2. Global composition of the scRNA-seq datasets. **a** Proportion of GEMs from mock and infected treatments from each dataset. **b** Proportion of bystanders, infected, mock, and infected mock cells in each dataset. **c** Distribution of cells with respect to viral loads for each treatment. Statistics presented in these plots correspond to observed counts. **d** Selecting cell with respect to number of individual transcripts, instead of number of detected genes increases the proportion of cells with high viral accumulations.

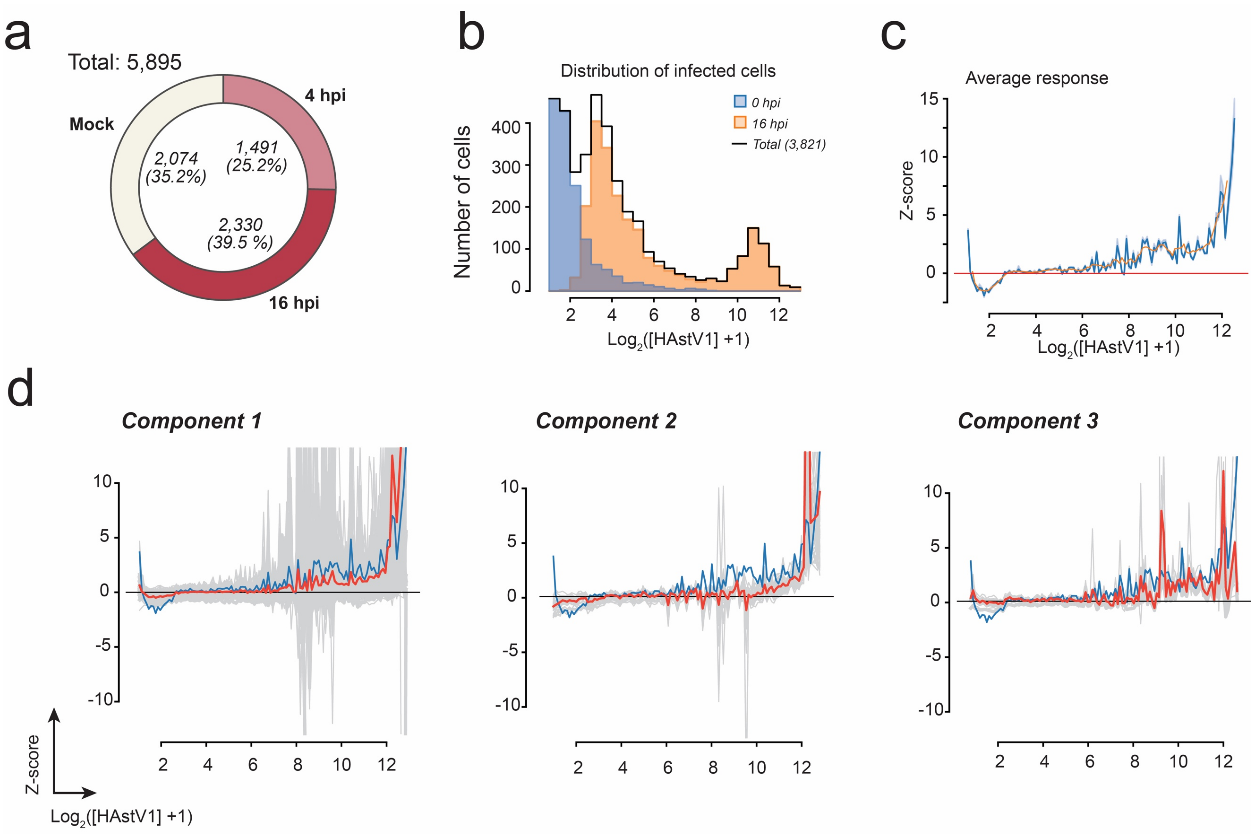

Supplementary Figure 3. Transcriptional response of ileum organoid cells infected with human astrovirus 1 (HAstV1). **a** Proportion of selected cells at each time post-infection. **b** Distribution of HAstV1 accumulations in the infected cell subset. **c** Average transcriptional response of ileum cells illustrating a very different response than SARS-CoV-2. **d** Response profiles of genes from components 1 (global SARS-CoV-2 response), 2 (genes involved in translation), and 3 (mitochondrial-encoded genes) to HAstV1 infection.

Supplementary Data 1. Database of reference human messenger RNAs and SARS-CoV-2 genomes used for mapping reads. Raw count matrices.

Supplementary Data 2. Expression matrices and transcript levels in uninfected cells.

Supplementary Data 3. Differential Expressed Gene analysis used in the volcano plots analysis. Datasets and results used in the Gene Ontology analyses. Classification of outliers.

Supplementary Data 4. Transcriptional profiles for all genes in each cell type.

Supplementary Data 5. Networks used in the analyses presented in Figs. 5 and 6.

Supplementary Data 6. Transcriptional analysis of human astrovirus 1 infection in ileum organoids.
