# Supplementary material for: Single-cell RNA-sequencing data analysis reveals a highly correlated triphasic transcriptional response to SARS-CoV-2 infection
Source: Commun Biol. 2022 Nov 27;5:1302. doi: 10.1038/s42003-022-04253-4 (PMC9701238; doi:10.1038/s42003-022-04253-4)
Supplement: Supplementary file 3 — Description of Additional Supplementary Files [file 42003_2022_4253_MOESM3_ESM.pdf]

Description of Additional Supplementary Files

File name: Supplementary Data 1

Description: Database of reference human messenger RNAs and SARS-CoV-2 genomes used for mapping reads. Raw count matrices.

File name: Supplementary Data 2

Description: Expression matrices and transcript levels in uninfected cells.

File name: Supplementary Data 3

Description: Differential Expressed Gene analysis used in the volcano plots analysis. Datasets and results used in the Gene Ontology analyses. Classification of outliers.

File name: Supplementary Data 4

Description: Transcriptional profiles for all genes in each cell type.

File name: Supplementary Data 5

Description: Networks used in the analyses presented in Figs. 5 and 6.

File name: Supplementary Data 6

Description: Transcriptional analysis of human astrovirus 1 infection in ileum organoids.
